# Regional Targeted Subcutaneous Injection of Botulinum Neurotoxin Type A in Refractory Chronic Migraine: A Randomized, Double-Blind, Placebo-Controlled Study

**DOI:** 10.3390/toxins15050324

**Published:** 2023-05-09

**Authors:** Francesco Bono, Maria Rosaria Mazza, Giuseppe Magro, Giorgio Spano, Giovanni Idone, Vincenzo Laterza, Denise Tedeschi, Francesco Pucci, Antonio Gambardella, Alessia Sarica

**Affiliations:** 1 Headache Center, and Center for Botulinum Toxin Therapy, Neurology Unit, Azienda Ospedaliero-Universitaria “Mater Domini”, 88100 Catanzaro, Italy; 2 Neurology Unit, Department of Medical and Surgical Sciences, Magna Graecia University of Catanzaro, 88100 Catanzaro, Italy; 3 Neuroscience Research Center, Department of Medical and Surgical Sciences, Magna Graecia University of Catanzaro, 88100 Catanzaro, Italy

**Keywords:** chronic migraine, subcutaneous BoNT-A injection paradigm, SjBoT injection paradigm, botulinum toxin type A, migraine treatment, follow the origin of maximum pain, migraine, OnabotulinumtoxinA

## Abstract

In this randomized, double-blind, placebo-controlled study, we evaluated the efficacy of an individualized technique of subcutaneous injection of botulinum toxin type A (BoNT-A) targeted (SjBoT) to the occipital or trigeminal skin area in non-responder patients with chronic migraine (CM). Patients who had not previously responded to at least two treatments of intramuscular injections of BoNT-A were randomly assigned (2:1) to receive two subcutaneous administrations of BoNT-A (up to 200 units) with the SjBoT injection paradigm or placebo. Following the skin area where the maximum pain began, treatment was given in the trigeminal or occipital region bilaterally. The primary endpoint changed in monthly headache days from baseline to the last 4 weeks. Among 139 randomized patients, 90 received BoNT-A and 49 received placebo, and 128 completed the double-blind phase. BoNT-A significantly reduced monthly headache days versus placebo (−13.2 versus −1.2; *p* < 0.0001) in the majority of patients who had cutaneous allodynia. Other secondary endpoints, including measures for disability (Migraine Disability Assessment questionnaire from baseline 21.96 to 7.59 after treatment, *p* = 0.028), also differed. Thus, in non-responder patients with CM, BoNT-A significantly reduced migraine days when administered according to the “follow the origin of maximum pain” approach using SjBoT injection paradigm.

## 1. Introduction

Chronic migraine (CM) is a disabling neurological disorder affecting 1.4–2.2% of the world’s population [1]. It is defined as a headache that occurs for 15 or more days per month for more than 3 months [2]. Overuse of analgesics, and psychiatric and sleep disorders, are common in these patients [3]. Treating these patients is therefore difficult, and a proportion of them do not respond or are intolerant to evidence-based preventive medications [4]. An additional therapeutic approach to treat these patients with CM who become drug-resistant is the use of intramuscular injections of botulinum toxin type A (BoNT-A) into the muscles of the cranial district [5,6,7,8].

However, as many as 30.4% of CM patients do not respond to treatment with intramuscular injections of BoNT-A, and many also report that they are not satisfied with the treatment [9]. To improve the response to BoNT-A injections in clinical practice, 57% of clinicians claimed to make variations in the intramuscular injection pattern of BoNT-A in patients with CM [10]. Some authors have also tested—in open-label pilot studies—other techniques of BoNT-A injection in migraine patients [11]. In addition, other authors have found that the efficacy of botulinum toxin injections into the pericranial and cranial muscles is not significantly different from that of placebo injections in migraine patients [12,13]. On the other hand, a growing body of evidence has demonstrated the antinociceptive action of botulinum toxin in chronic neuropathic pain [14,15]. In that condition, the anatomical substrate of the peripheral analgesic action of BoNT-A was its binding to cutaneous sensory or nociceptive nerve endings, where it blocked the release of several neurotransmitters. In fact, patients with lower cutaneous sensory fiber density had a poor response to subcutaneous injections of BoNT-A [16]. Considering that the density of sensory/nociceptive nerve endings is elevated in the cranial skin, and because of cutaneous sensory or nociceptive signals from the pericranial tissues via the pericranial nerves also reach the trigeminal spinal nucleus [17], we speculated that the antinociceptive action of botulinum toxin in CM could therefore be enhanced by subcutaneous administration into the skull. On this basis, we hypothesized that the “Follow the Origin of Maximum Pain” approach using subcutaneous injections of BoNT-A targeted (SjBoT) to regional occipital or trigeminal regions could represent an effective injection strategy in non-responsive patients with CM. To test the efficacy of this new BoNT-A injection paradigm, we treated patients with CM who had not responded to intramuscular injections of BoNT-A with the SjBoT paradigm in the pericranial/cranial muscles.

## 2. Results

The characteristics of the 139 patients with CM enrolled in this study are summarized in Table 1.

**Figure 1:**
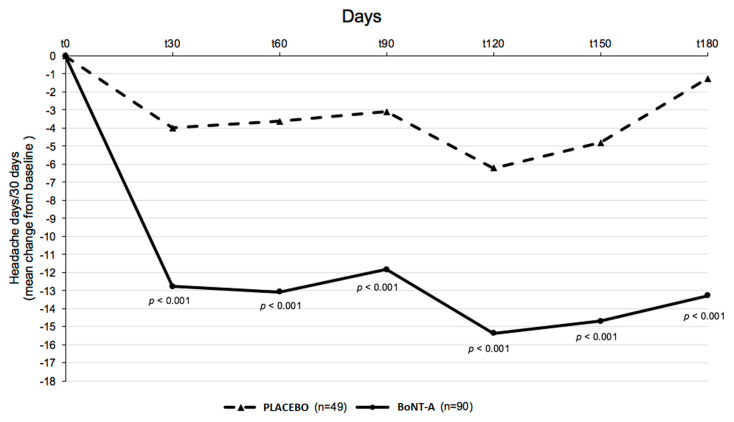
Mean change from baseline in frequency of headache days in the placebo and BoNT-A group treated with SjBoT paradigm.

**Figure 2:**
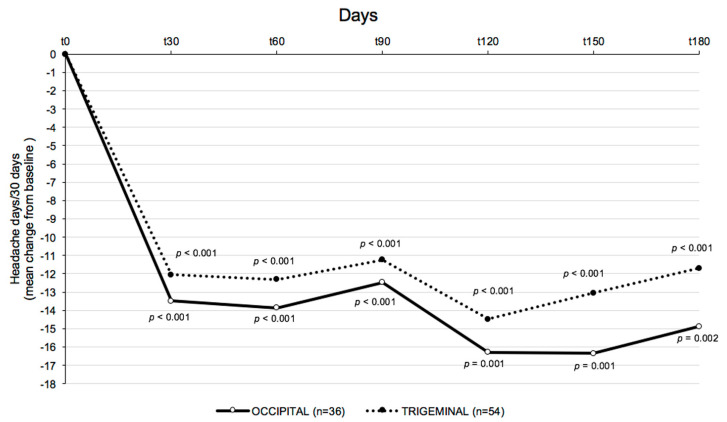
Mean change from baseline in frequency of headache days in the occipital and trigeminal group after BoNT-A treatment with SjBoT paradigm.

In detail, 54 patients underwent BoNT-A injections in the trigeminal skin area and 36 patients in the occipital skin area. In contrast, 49 patients underwent placebo treatment in the trigeminal or occipital skin area. The reduction in monthly headache days between BoNT-A treatment and placebo was −13.2 versus −1.2 days, *p* < 0.0001. The reduction in monthly headache days was −11.6 in the trigeminal group versus −14.8 in the occipital group (*p* < 0.001) in the BoNT-A treatment. (Figure 1 and Figure 2).

During follow-up, differences were found in HAMA and BDI-II scores compared with baseline in patients undergoing BoNT-A treatment; in the trigeminal group, BDI-II scores went from 12.65 ± 9.55 to 9.94 ± 9.93 (*p* = 0.048), HAMA scores went from 10.56 ± 11 to 8.14 ± 6.46 (*p* = 0.048). On the other hand, HARS scores went from 18.56 ± 12.49 at baseline to 15.76 ± 12.16 (*p* = 0.952) 180 days after treatment in the trigeminal group; in the occipital group, HARS scores went from 19.55 ± 12.7 to 14.17 ± 12.28 (*p* = 0.080). A significant difference in pain intensity and migraine-related disability was observed in the BoNT-A treatment group: in the trigeminal group, the VAS score went from 9.09 ± 1.46 at baseline to 8.21 ± 2.22 at 180 days (*p* = 0.011); in the occipital group, the VAS score went from 8.86 ± 1.78 to 6 ± 2.69 (*p* = 0.007). As for migraine-related disability, MIDAS went from 21.96 ± 16.32 at baseline to 7.59 ± 6.24 after treatment (*p* = 0.028) in the trigeminal group. On the other hand, in the occipital group, MIDAS went from 29.4 ± 22.19 to 8.14 ± 6.47 (*p* = 0.001) (Table 2). Adverse events seen in the BoNT-A treatment group were modest eyelid weakness that regressed after a few months in three patients (3.3%) and modest burning at the time of subcutaneous injection; in contrast, no major adverse events were reported in the placebo treatment, except for modest burning at the time of subcutaneous injection.

## 3. Discussion

This is the first randomized, double-blind, placebo-controlled study demonstrating the efficacy and safety of the “follow the origin of maximum pain” approach using subcutaneous injections of BoNT-A targeted (SjBoT) to the occipital or trigeminal skin area in non-responder patients with chronic migraine. Our results show that subcutaneous injections of BoNT-A targeted to the skin area of maximum headache pain origin significantly reduce migraine frequency, pain severity, and disease disability.

The efficacy of the individualized SjBoT injection paradigm is based on the antinociceptive effect of subcutaneous injections of BoNT-A and on the targeted regional administration of BoNT-A using the “follow the origin of maximum pain” approach.

Consistent with our results, several animal studies confirmed the antinociceptive effects of subcutaneous injection of botulinum toxin type A [18,19]. In comparison, others demonstrated the effects of subcutaneous injection of BoNT-A on neuropathic pain disorders, testing the hypothesis that BoNT-A can block nociceptor transduction [16,20]. BoNT-A targets receptors such as TRPV1 (transient receptor potential cation channel subfamily V member 1), whose insertion into the lipid bilayer of the synaptic membrane is critical for proper pain signaling [21,22]. Indeed, a randomized, double-blind, placebo-controlled trial showed that repeated subcutaneous injections of BoNT-A in 152 patients with peripheral neuropathic pain had a sustained analgesic effect [16]. The patients who responded better to toxin were those who had a higher intra-epidermal nerve fiber density. This fact may explain why a diffuse subcutaneous injection paradigm led to the best outcomes in those patients with peripheral neuropathic pain.

In the context of migraine, extracranial application of BoNT-A was shown to inhibit mechanical transduction in the suture branches of meningeal nociceptors, thus inhibiting mechanical nociception in peripheral trigeminovascular neurons [20,23]. The anatomical substrate of the analgesic action of BoNT-A on migraine may be due to the binding to the peripheral nociceptive nerve endings of extracranial tissues where it inhibits SNARE-dependent regulated exocytosis of proinflammatory and excitatory neurotransmitters and neuropeptides release and thus blocks the activation of extracranial branches of intracranial nociceptors [24,25]. The data are supported by recent studies that showed a link between intra and extracranial nerve structures; in fact, in rats, BoNT-A injections near extracranial nerve endings of suture branches of intracranial meningeal nociceptors reduced the sensitivity of these nociceptors [26]. This fact indicates that the number of extracranial nerve endings reached by the toxin might be one of the factors responsible for the analgesic action of BoNT-A in migraine and explains why extensive subcutaneous administration of BoNT-A achieves a better antinociceptive effect in patients treated with the SjBoT injection paradigm.

Another important contributing factor to the efficacy of the SjBoT injection paradigm for the prevention of CM is the regional administration of BoNT-A in the cutaneous area of the maximum pain’s origin. The latter is the area of ictal cutaneous allodynia during migraine attack, which represents the clinical expression of peripheral nociceptor sensitization [27]. Higher frequency and longer duration of migraine attacks have been shown to correlate with the development of central sensitization and higher incidences of cutaneous allodynia [27,28]. Previous studies have shown that BoNT-A inhibits peripheral sensitization, leading to an indirect reduction in central sensitization [29]. A new possible explanation of this effect may be that BoNT-A also reduces high numbers of immune cells in the calvarial periosteum of headache patients [25]. Thus, BoNT-A can also help reduce pre-existing localized inflammation, one of the factors involved in the pathogenesis of headache pain. Together, these data explain the efficacy of the SjBoT injection paradigm in the majority of allodynic patients with CM independently from trigeminal or occipital treatment and the changes of cutaneous allodynia during the follow up of the patients. Our findings indicate that the skin area of maximum cephalalgic pain, which corresponds to the site of ictal cutaneous allodynia, is the target for subcutaneous injections of BoNT-A in patients with CM.

The strength of this pilot study is intrinsically linked to its design. Another important finding is that the individualized SjBoT injections paradigm appears to be a safe treatment. The limitation is that this is a single-center study, and other limitations are due to the small number of patients, the lack of evaluation of long-term effects of repeated administration of the treatment, and the administration of a single placebo treatment. However, in view of the severity of migraine in these patients and the poor response to placebo treatment, it was appropriate to administer a single dose of placebo. Another possible limitation is that the placebo response was low compared with what was observed in the PREEMPT studies [30,31]. This difference in placebo response may be due to the different methods of placebo preparation. In our case, the placebo consisted of saline prepared in a syringe indistinguishable from the syringe with BoNT-A, whereas the PREEMPT studies used a true placebo. However, our placebo preparation was the one validated in the BOTNEP study for the treatment of neuropathic pain [16].

In conclusion, the data from this study demonstrate that the “Follow the Origin of Maximum Pain” approach using the individualized SjBoT paradigm may be a useful option for the preventive treatment of CM in non-responder patients and suggests the need to do further similar studies to confirm our results.

## 4. Materials and Methods

This is a single-center, double-blinded, randomized, placebo-controlled interventional trial conducted at a tertiary Headache Centre of the academic hospital in Catanzaro, Italy.

### 4.1. Ethics Committee

Prior to inclusion in the study, written informed consent was obtained from all patients, and the study was approved by the local Ethics Committee according to the Declaration of Helsinki.

### 4.2. Inclusion Criteria

(i) Men or women between 18 and 50 years of age with CM, satisfying the diagnostic criteria of the International Classification of Headache disorders (ICHD-3) [2];
(ii) Failure of at least two treatments with intramuscular injections of BoNT-A in the cranial/pericranial muscles performed more than 6 months ago;
(iii) For women of child-bearing potential, the use of highly effective contraception.

Overuse of drugs was defined as taking simple analgesics for ≥ 15 days, or other types of drugs or combinations of types for ≥ 10 days.

### 4.3. Exclusion Criteria

(i) Presence of diseases interfering with neuromuscular function;
(ii) Presence of psychiatric disorder;
(iii) Other primary/secondary headaches.

Tension-type headache was allowed if the patient could clearly distinguish the two types of attack.

### 4.4. Endpoints

The primary endpoint was considered the change in monthly headache days. Secondary outcomes were: change in Migraine Disability Assessment (MIDAS) questionnaire, Hamilton Anxiety Rating Scale (HARS), Beck Depression Inventory-II (BDI-II) scores, change in pain intensity, all compared with baseline.

### 4.5. Study Design 

**Figure 3:**
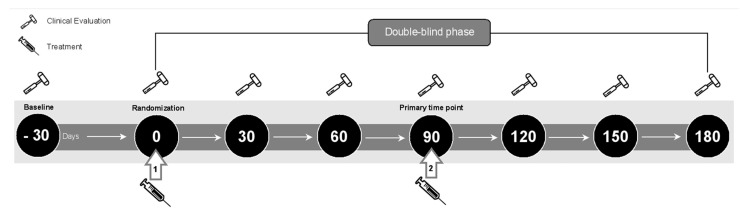
Subcutaneous injections of BoNT-A-targeted (SjBoT) study design.

At baseline, each patient provided a diary of headache days in the previous month. All patients underwent thorough clinical data collection to identify the skin area of onset of maximum headache pain and the characteristics of headache. The 12-item Cutaneous Allodynia Symptom Checklist (ASC-12) was administered during a headache attack. Pain and disability were assessed using the Visual Analog Scale (VAS) from 0 to 10, and the MIDAS questionnaire. Mood and anxiety were assessed with the BDI-II and the HARS scores. At follow-up, each patient was evaluated every 30 days until 180 days after treatment. The patient reported headache day data for the previous month and underwent the same tests completed at baseline evaluation. All patients were allowed to use medication for the acute migraine attack and preventive therapy at the same levels as before baseline, but other methods of pain control or new preventive treatments were prohibited. The principal investigator, who was not involved in treating and assessing the outcome of patients, randomized the eligible patients into BoNT-A treatment or placebo group in a 2:1 ratio; the therapist administered the treatment in the outpatient clinic; the investigator who assessed the outcome avoided physical contact between patients during the treatment; the patients were instructed to fill in diaries recording headache occurrence before treatment and during months post-treatment. The investigator who assessed outcome, the patients, and the therapist were all blinded. The blinded investigator created the database from the headache diaries filled by the patients. Patients were randomized to BoNT-A or placebo treatment (Figure 3), then patients were grouped in trigeminal or occipital treatment according to skin area of origin of maximum pain. In addition, based on the severity scores of the ASC-12 CA, patients were grouped into a non-allodynic group (no or mild cutaneous allodynia with a score between 0 and 5) or an allodynic group (moderate or severe cutaneous allodynia with a score above 6). An operator, who was not involved in any other aspect of the study, prepared the syringes used for injections of the appropriate treatments, so that the physicians performing the injections would not recognize the treatment. The syringes, active treatment, and placebo solutions were transparent and indistinguishable. All patients and investigators were masked for treatment assignment.

### 4.6. The “Follow the Origin of Maximum Pain” Approach

**Figure 4:**
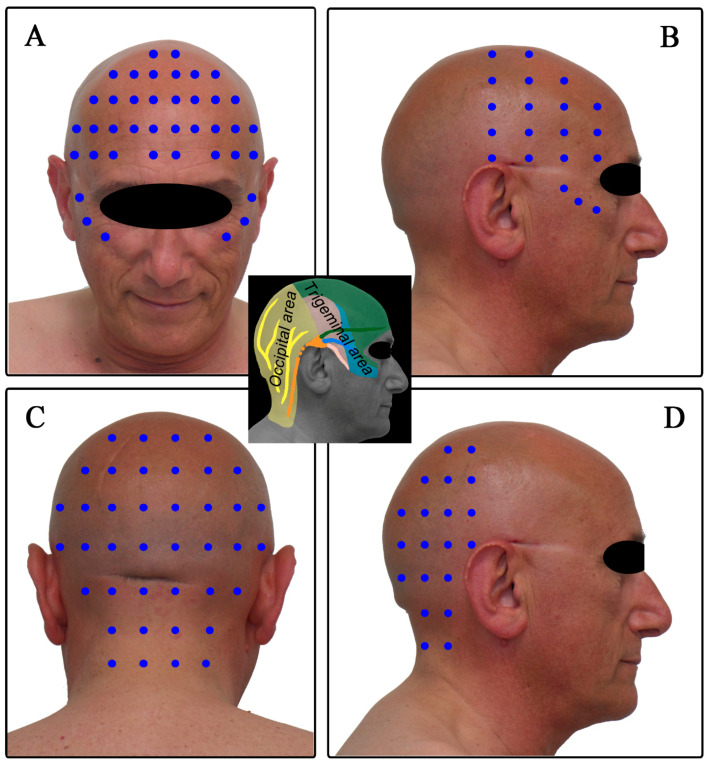
The “follow the origin of maximum pain” approach: based on the skin area of origin of maximum pain, patients were assigned to trigeminal or occipital treatment with BoNT-A (central panel). SjBoT injection paradigm: the skin sites of the trigeminal region injected with BoNT-A (**A**,**B**); the skin sites of the occipital region injected with BoNT-A (**C**,**D**).

This approach consisted of regional administration of BoNT-A into the skin area of origin of maximum pain in patients with CM. The skin area of maximum cephalalgic pain was thus the target of subcutaneous injections of ONA, and according to this feature, patients were assigned to the trigeminal or occipital treatment with SjBoT paradigm (Figure 4).

### 4.7. The SjBoT Injection Paradigm

The BoNT-A (onabotulinum) and placebo treatment consisted of between 20 and 40 subcutaneous injection sites placed at a mutual distance between 1.5 cm and 2.0 cm into the skin area of onset of maximum pain according to the SjBoT injection paradigm: the skin area innervated by the first branch and a small part of the second and third branch of the trigeminal nerve for treatment of the trigeminal region (Figure 4A,B); the skin area innervated by the great, lesser occipital nerves for treatment of the occipital region (Figure 4C,D). Each 100 U vial of onabotulinumA (Botox, Abbvie) was dissolved in 2 mL of 0.9% sodium chloride, resulting in a concentration of 5U/0.1 mL. Five units (0.1 mL) were injected subcutaneously at each site (Figure 4). Needle size: 30 gauges, 1.3 cm length. The BoNT-A treatment consisted of 20–40 bilateral subcutaneous injections of BoNT-A (up to 200 units) into the trigeminal or occipital skin areas. The BoNT-A treatment was repeated after 90 days. The placebo treatment consisted of 20–40 subcutaneous injections of 0.1 mL of 0.9% sodium chloride into the trigeminal or occipital skin areas and was not given to the patient a second time. Subcutaneous injections were administered following the same protocol as the BoNT-A group and the placebo group.

### 4.8. Statistical Analysis

Statistical analysis was performed using Statistical Package for Social Science software (SPSS, v20.0, Chicago, IL, USA) for Macintosh. Differences in frequency distributions between patients in the BoNT-A treatment and placebo groups, separately for patients in the trigeminal and occipital groups—of (i) sex and (ii) allodynic and non-allodynic patients—were assessed using the Chi-square test. Regarding demographic and clinical data, the comparison of patients in the BoNT-A and placebo group was separate for patients treated in the trigeminal or occipital skin area at baseline and post-treatment (t180) using (i) the two-sample unpaired *t*-test for age and BMI and (ii) the Mann–Whitney test for BDI, HARS, VAS, and MIDAS. Differences between baseline and post-treatment (t180) in the BoNT-A group—separately for patients in the trigeminal and occipital group—of BDI, HARS, VAS, and MIDAS were assessed with a single-tailed Wilcoxon rank-sum test. Evaluation of differences in the primary endpoint, i.e., the reduction in the number of headache days per month and mean change in total headache days per month, between patients in the BoNT-A group and those in the placebo group, separately for trigeminal and occipital patients, were performed with Fisher’s exact test and the Mann–Whitney test. The change in the secondary endpoint at each time point, t30, t60, t90, t120, t150, t180, compared with baseline in the BoNT-A treatment (trigeminal and occipital) groups were analyzed with a single-tailed Wilcoxon test. A statistically significant result was considered if *p* < 0.05, and when not explicitly specified, all *p*-values were two-tailed.

## Figures and Tables

**Table 1:** Demographics and characteristics of the 139 patients with chronic migraine.

Patients	
Age, years, mean ± SD	35 ± 10.2
Sex, F/M	119/20
Body mass index, kg/m2, mean ± SD	25 ± 4
Duration, years, mean ± SD	14 ± 9
**Headache Profile, (%)**	
Unilateral headache	71%
Pulsating pain	71%
Severity of pain	
Severe	69%
Cutaneous area where pain started	
Trigeminal	60%
Occipital	40%
Frequency of headache	
Daily	79%
Overuse medication	40%
**ASC 12 Cutaneous Allodynia (Allodynic Score ≥ 6), (%)**	
Allodynic patients	60%
Trigeminal	81.5%
Occipital	31%
**Patient’s Score at Baseline**	
MIDAS mean ± SD	25 ± 18
BDI-II mean ± SD	12 ± 10
HARS mean ± SD	19 ± 12
VAS mean ± SD	9 ± 1

Abbreviations: F, female; M, male; ASC-12, validated 12-item allodynia symptom checklists; CA, cutaneous allodynia; AS, visual analog scale; MIDAS, migraine disability assessment; BDI II, Beck Depression Inventory II; HARS, Hamilton Anxiety Rating Scale.

**Table 2:** Effects of BoNT-A or placebo treatment with the SjBoT paradigm on secondary endpoints in chronic migraine patients.

Patients (*n*)	Trigeminal Group 88			Occipital Group 51		
	BoNT-A	PLACEBO	*p*	BoNT-A	PLACEBO	*p*
Age, years, mean ± SD	39.92 ± 10.35	37.79 ± 11.4	0.37	42.91 ± 12.36	45.73 ± 16.20	0.5
Sex, F/M	51/3	30/4	0.42	27/9	11/4	1
Body mass index, kg/m^2^, mean ± SD	25.05 ± 4.59	24.8 ± 5.72	0.82	24.51 ± 3.19	25.7 ± 3.64	0.27
Allodynic patients	43 ^i^	25	0.60	11 ^i^	8	0.34
Non-allodynic patients	11	9	0.60	25	7	0.34
BDI II baseline (mean ± SD)	12.65 ± 9.55 ^a^	9.47 ± 4.85	0.45	10.56 ± 11 ^e^	10.68 ± 5.57	0.14
BDI II post-treatment (mean ± SD)	9.94 ± 9.93	9.53 ± 6.01	0.57	8.14 ± 6.46	10.72 ± 6.36	0.089
HARS baseline (mean ± SD)	18.56 ± 12.49 ^b^	14.95 ± 0.09	0.38	19.55 ± 12.7 ^f^	15.60 ± 9.78	0.83
HARS post treatment (mean ± SD)	15.76 ± 12.16	14.70 ± 9.74	0.93	14.17 ± 12.28	15 ± 9.87	0.75
VAS baseline (mean ± SD)	9.09 ± 1.46 ^c^	8.86 ± 1.50	0.34	8.86 ± 1.78 ^g^	8.78 ± 1.45	0.31
VAS post treatment (mean ± SD)	8.21 ± 2.22	7.65 ± 1.95	0.24	6 ± 2.69	7.85 ± 1.85	0.061
MIDAS baseline (mean ± SD)	21.96 ± 16.32 ^d^	30.71 ± 56.74	0.94	29.4 ± 22.19 ^h^	28.7 ± 50.41	0.46
MIDAS post treatment (mean ± SD)	7.59 ± 6.24	29.9 ± 58.14	<0.001	8.14 ± 6.47	28 ± 51.31	0.016

SjBoT denotes subcutaneous injections of BoNT-A targeted; ^a^ *p* = 0.048 compared to post-treatment; ^b^ *p* = 0.952 compared to post-treatment; ^c^ *p* = 0.011 compared to post-treatment; ^d^ *p* = 0.028 compared to post-treatment; ^e^ *p* = 0.093 compared to post-treatment; ^f^ *p* = 0.080 compared to post-treatment; ^g^ *p* = 0.007 compared to post-treatment; ^h^ *p* = 0.001 compared to post-treatment; ^i^ *p* = 0.001 comparison of the distribution of cutaneous allodynia between trigeminal and occipital groups.

## Data Availability

Not applicable.
